# Tuberculosis and latent infection in employees of different prison unit types

**DOI:** 10.11606/S1518-8787.2018052007127

**Published:** 2018-01-29

**Authors:** Péricles Alves Nogueira, Regina Maura Cabral de Melo Abrahão, Vera Maria Neder Galesi, Rossana Verónica Mendoza López

**Affiliations:** I Universidade de São Paulo. Faculdade de Saúde Pública. Departamento de Epidemiologia. São Paulo, SP, Brasil; II Governo do Estado de São Paulo. Secretaria de Estado da Saúde. Centro de Vigilância Epidemiológica “Prof. Alexandre Vranjac”. São Paulo, SP, Brasil; III Universidade de São Paulo. Faculdade de Medicina. Instituto do Câncer do Estado de São Paulo Octávio Frias de Oliveira. Centro de Investigação Translacional em Oncologia. São Paulo, SP, Brasil

**Keywords:** Tuberculosis, epidemiology, Latent Tuberculosis, diagnosis, Prisons, Employees, Risk Factors, Working Conditions, Occupational Health, Tuberculose, epidemiologia, Tuberculose Latente, diagnóstico, Prisões, recursos humanos, Fatores de Risco, Condições de Trabalho, Saúde do Trabalhador

## Abstract

**OBJECTIVE:**

Estimate the prevalence of active tuberculosis and latent tuberculosis infection among the staff that is in contact and the staff that is not in contact with prisoners, and investigate factors associated with latent tuberculosis infection in this population.

**METHODS:**

Observational cross-sectional study, conducted from 2012 to 2015, in employees of different prison units in the municipality of Franco da Rocha, SP. It consisted of the application of a questionnaire, application and reading of the tuberculin test, sputum smear microscopy, sputum culture, and radiological examination. The association between the qualitative variables was calculated by the Pearson's chi-squared test. The sociodemographic and clinical-epidemiological factors related to the latent tuberculosis infection were evaluated by the logistic regression with the odds ratios (OR) calculation and their respective intervals with 95% of confidence (95%CI).

**RESULTS:**

A total of 1,059 employees were examined, 657 (62.0%) of prisons, 249 (23.5%) of CASA Foundation units and 153 (14.5%) of custodial and psychiatric treatment hospitals. The tuberculin test was applied and read for 945 (89.2%) professionals. Of these, 797 (84.3%) were contacts of detainees and 148 (15.7%) were not. Among prison staff, the factors associated with latent tuberculosis infection were: contact with detainee (OR = 2.12, 95%CI 1.21–3.71); male gender (OR = 1.97, 95%CI 1.19–3.27); between 30 and 39 years old (OR = 2.98, 95%CI 1.34–6.63), 40 to 49 years old (OR = 4.32, 95%CI 1.94–9.60), and 50 to 59 years old (OR = 3.98, 95%CI 1.68–9.43); nonwhite color or race (OR = 1.89, 95%CI 1.29–2.78); and smoker (OR = 1.64, 95%CI 1.05–2.55). There were no positive test on sputum smear microscopy and culture. Of the 241 (22.8%) professionals who underwent radiological examination, 48 (19.9%) presented alterations of which 11 were suspected of tuberculosis.

**CONCLUSIONS:**

Prison employees who have direct contact with detainees are 2.12 times more likely to become infected with *Mycobacterium tuberculosis* in the work environment and consequently to become ill with tuberculosis and should be targeted for disease prevention and control.

## RESUMO

**OBJETIVO:**

Estimar a prevalência de tuberculose ativa e de infecção latente da tuberculose entre funcionários contatos e não contatos de detentos, e investigar fatores associados à infecção latente da tuberculose nesta população.

**MÉTODOS:**

Estudo observacional do tipo transversal, realizado no período de 2012 a 2015, em funcionários de diferentes unidades prisionais do município de Franco da Rocha, SP. Consistiu na aplicação de um questionário, aplicação e leitura da prova tuberculínica, baciloscopia e cultura dos escarros e exame radiológico. A associação entre as variáveis qualitativas foi calculada pelo teste qui-quadrado de Pearson e os fatores sociodemográficos e clínico-epidemiológicos relacionados à infecção latente da tuberculose foram avaliados pela regressão logística com o cálculo das *odds ratios* (OR) e seus respectivos intervalos com 95% de confiança (IC95%).

**RESULTADOS:**

Foram examinados 1.059 funcionários, sendo 657 (62,0%) de penitenciárias, 249 (23,5%) de unidades da Fundação CASA e 153 (14,5%) de hospitais de custódia e tratamento psiquiátrico. Foi aplicada e lida a prova tuberculínica em 945 (89,2%) profissionais. Desses, 797 (84,3%) eram contatos de detentos e 148 (15,7%) não eram. Entre funcionários das penitenciárias, os fatores associados com a infecção latente da tuberculose foram os seguintes: ter contato com detento (OR = 2,12; IC95% 1,21–3,71); ser do sexo masculino (OR = 1,97; IC95% 1,19–3,27); estar na faixa etária entre 30 e 39 anos (OR = 2,98; IC95% 1,34–6,63), 40 a 49 anos (OR = 4,32; IC95% 1,94–9,60) e 50 a 59 anos (OR = 3,98; IC95% 1,68–9,43); ser da cor ou raça não branca (OR = 1,89; IC95% 1,29–2,78); e ser fumante (OR = 1,64; IC95% 1,05–2,55). Não houve exame positivo na baciloscopia e na cultura. Dos 241 (22,8%) profissionais que realizaram o exame radiológico, 48 (19,9%) apresentaram alterações, dos quais 11 eram suspeitos de tuberculose.

**CONCLUSÕES:**

Os funcionários das penitenciárias que têm contato direto com os detentos têm 2,12 vezes mais chance de se infectar pelo *Mycobacterium tuberculosis* no âmbito de trabalho e, consequentemente, de adoecer por tuberculose, devendo ser alvos de ações de prevenção e controle da doença.

## INTRODUCTION

Work health considers tuberculosis (TB) a difficult problem to be controlled in closed places such as prison environments because it is a disease of respiratory transmission. Agglomeration, lack of ventilation and natural light are common in most of the country's criminal units^9^ ^,^ ^10^ ^,^ ^15^ ^,^ ^24^ ^,^ ^29^ ^,^ ^a^ ^,^ ^b^ ^,^ ^c^. According to the Ministry of Health the main diseases identified in Brazil's prisons are: TB, sexually transmitted diseases (STD), hepatitis and dermatoses^d^. In absolute numbers, Brazil has the fourth largest prison population worldwide, behind only the United States, China, and Russia, with 607,731 detainees in June 2014, distributed in 1,424 prison units^e^. The Department of Penitentiary Administration of the State of São Paulo^f^ has 165 prison units distributed throughout the state and the State Justice and Citizenship Defense Agency has 149 socio-educational centers, called the Fundação Centro de Atendimento Socioeducativo ao Adolescente (CASA Foundation)^g^.

Of the total number of Brazilian prisoners, 36% (219,053) are in the state of São Paulo and the TB incidence rates in this population reach about 800 cases per 100,000 inmates, well above 39.1 cases per 100,000 inhabitants in the general population of the State^21^ ^,^ ^e^. In 2014, about 12% of TB cases in the state occurred in the prison population^12^.

Studies conducted since the late 1990s have confirmed the high rate of TB transmission in prisons in both developed and developing countries and observed a much higher prevalence and incidence of TB in the prison population than in the general population^2^ ^,^ ^4^ ^,^ ^5^ ^,^ ^10^ ^,^ ^15^ ^,^ ^25^ ^,^ ^26^ ^,^ ^h^. As a result the WHO proposed that measures to control TB transmission be adopted in so-called risk environments, that is, places that offer a high chance of infection by tuberculosis bacilli^7^ ^,^ ^8^ ^,^ ^24^.

In Brazil, the incidence of the disease in the penitentiary system is 28 times higher than in the general population^17^. Health conditions in Brazilian prisons are degrading and transform detainees into an important reservoir of TB bacillus and, consequently, into sources of infection when released^1^ ^,^ ^3^ ^,^ ^4^ ^,^ ^10^ ^,^ ^11^ ^,^ ^22^ ^,^ ^c^. In addition, infectious-contagious diseases are not restricted to prisons, since many are taken into society by the contingent of about 200,000 prison staff who have direct contact with the prison population, by the prisoners’ relatives and most importantly by intimate visits^2^ ^,^ ^10^ ^,^ ^17^ ^,^ ^d^.

Therefore, inmates and workers of the penal system are exposed to numerous health risks and may be considered as members of populations vulnerable to various pathologies^2^ ^,^ ^9^ ^,^ ^24^ ^,^ ᵃ ^,^ ^i^. TB can be referred to an occupational disease par excellence in the health area with documented cases of the transmission from prisoners to employees^2^ ^,^ ^9^ ^,^ ^j^. However, there is no specific occupational health program in relation to TB aiming at the protection of professionals in the prison system who have high infection rates and risk of becoming ill due to this pathology.

In view of this context, the objective of this study was to estimate the prevalence of active tuberculosis and latent tuberculosis infection among the staff in contact and not in contact with prisoners and to investigate factors associated with latent tuberculosis infection in this population.

## METHODS

Since May 2011 the Epidemiological Surveillance Center of the State Health Department of São Paulo together with the Health Department of the Penitentiary Administration Department and the Epidemiology Department of the Public Health School of the Universidade de São Paulo, has been carrying out, under the auspices of the World Bank a study entitled “Tuberculinic survey of the prison system in the metropolitan area of São Paulo” aiming at the elaboration of a proposal of occupational health in tuberculosis for professionals of this system.

This study was conducted only in prisons in the greater São Paulo area. Preliminarily, it was observed that the employees who had direct contact with the inmates presented greater reactivity to the tuberculin test. That is, they were infected with *Mycobacterium tuberculosis*. It was not known whether employees of other types of prison units had the same behavior in relation to latent tuberculosis infection (LTBI).

As the municipality of Franco da Rocha had 12 prison units including: three prisons, one Provisional Detention Center and one Penitentiary Progression Center belonging to the Department of Penitentiary Administration plus five CASA Foundation units and two custodial and psychiatric treatment hospitals, it was elected as the ideal place to understand the magnitude of active pulmonary TB and LTBI in prison system servers and all civil servants hereinafter referred to as “employees”.

The penitentiaries are places where the prisoners, already tried, serve their sentence in a closed regime. The Provisional Detention Center houses provisional prisoners awaiting trial in a closed regime. The Penitentiary Progression Center is a prison unit built next to closed regime establishments, for prisoners in the semi-open regime^f^. The CASA Foundation units aid young offenders between the ages of 12 and 21 who are included in the socio-educational measures of deprivation of liberty (internment) and semi-liberty throughout the state of São Paulo, whose release is compulsory at age 21^g^. Custody hospitals, former judicial asylums, harbor individuals judged and considered unimpeachable, that is, who are unable to answer for their own acts^f^.

A cross-sectional observational study between 2012 and 2015 was conducted in 1,059 employees from the 12 prison units in the municipality of Franco da Rocha, where the presence of latent tuberculosis infection was evaluated simultaneously with respect to the study variables and in relation to the characteristics of the population.

The following criteria were used to select the cases of active pulmonary TB: any employee with a diagnosis confirmed by sputum smear microscopy or culture and one in which the physician, based on clinical-epidemiological data and the result of complementary tests, such as radiological examination, affirms the diagnosis of TB. As a case of LTBI we considered every employee who underwent the tuberculin test whose tuberculin was PPD-RT 23, applied intradermally in the middle third of the left anterior forearm at the dose of 0.1 ml, which contained two TUs (tuberculin units), and whose reading of the greatest transverse diameter of the indentation perpendicular to the forearm after 72 hours of application and performed with a transparent millimeter ruler resulted in an indentation ≥ 10 millimeters^k^ ^,^ ^l^.

Inclusion criteria in the study were voluntary adherence and attendance at all stages of the study. The exclusion criteria were the employee's refusal to participate in the study; non-attendance at any stage of the data collection, even if they had signed informed consent; and those who were on vacation or medical leave.

The variables analyzed were classified into two groups: a) sociodemographic: sex, age, marital status, color or race, level of education, and b) clinical-epidemiological: presence of cough, smoking, other lung disease, previous tuberculosis, contact with detainees, sputum smear microscopy, culture, chest radiography and tuberculin skin test.

When the research was approved by the Ethics Committees of the Faculdade de Saúde Pública of the Universidade de São Paulo and the State Penitentiary Administration Department, the multiprofessional work team was assembled and trained to collect data. At the same time a meeting took place with the directors and staff of the different prison units to clarify the procedures that would be carried out and the impact of tuberculosis in the prison system and to prepare a worksheet for them.

In the field, each employee who agreed to participate in the study and signed the free and informed consent form was interviewed, individually and quietly, by a member of the work team. In the interview a validated questionnaire was used^m^ which contained questions about sociodemographic and clinical-epidemiological data. The following information was entered: name, filiation, sex, age, marital status, color or race, level of education, time working in the prison unit, sector in which they worked, position held, direct contact with detainees, previous tuberculosis, previous contact with tuberculosis patients, presence of cough, expectoration, smoking, other lung disease, other non-pulmonary disease, and whether the tuberculin test had already been performed.

Next, nurses checked by the State Department of Health applied the tuberculin test (PPD-RT23 - 2 TU/0.1 ml), according to Ministry of Health^l^ regulations, to all participants. The employee who presented induration ≥ 10 mm was considered to be infected by *M. tuberculosis* or as reacting to the tuberculin skin test, according to recommendations of the Ministry of Health^k^ ^,^ ^l^ and the CDC^n^. The employees were divided into two groups: those with induration less than 10 mm, considered negative; and those with a measurement greater than or equal to 10 mm, the positive ones. They were also classified as “contacts” and “non- contacts” of detainees.

Employees who had direct contact with the detainees were classified as contacts, namely: prison security officers (PSO); health professionals; the teachers; religious agents; the general director and the director of security and discipline. Classified as non-contacts were those who had no direct contact with detainees such as: administrative officers; escort and prison surveillance agents (EPSA) and the employees of the board.

An active TB search was carried out in this population and a sputum sample was collected from each participant for sputum smear and culture examinations according to the recommendations of the Ministry of Health^k^. Positive cultures would be identified and tested for sensitivity to antituberculosis drugs.

Symptomatic respiratory employees detected by the individual questionnaire and reactors to the tuberculin test were referred for radiological examination of the chest and for a second sputum examination. The staff that presented abnormalities in the radiological examination was referred to the health units of the region.

Qualitative variables were presented by frequencies and percentages. The association between these variables was calculated by Pearson's chi-squared test or Fisher's exact test, according to frequency characteristics^19^. To evaluate the association between LTBI and the different independent variables, univariate and multiple logistic regression was used. Initially, odds ratios (OR) and their respective 95% confidence intervals (95%CI) were calculated for each variable. Those with p-value ≤ 0.20 were selected for multiple regression. The stepwise backward method allowed us to analyze the final logistic regression model with the independent variables that were statistically significant and the adjustment variable. The adjustment of the model was evaluated by the Hosmer-Lemeshow test, under the null hypothesis that “the model is good”^19^. The level of significance was 5%. The analyses were performed in the statistical software SPSS v.18 for Windows^®^ ^19^.

This study was approved by the Research Ethics Committees of the Faculdade de Saúde Pública of the Universidade de São Paulo (Report 548.121/2014) and the State Department of Penitentiary Administration (Report 019/2011). Participation was voluntary, and all employees signed a free and informed consent form.

## RESULTS

At the time of the survey there were 2,143 employees in the 12 prison units in the municipality of Franco da Rocha distributed as follows: 720 (33.6%) in the three penitentiaries; 243 (11.3%) in the Provisional Detention Center; 297 (13.9%) in the Penitentiary Progression Center; 511 (23.8%) in the two custodial and psychiatric treatment hospitals and 372 (17.4%) in the five CASA Foundation units. Of these, 1,059 (49.4%) were present and accepted to participate in the study.

Of the 1,260 employees of the five units of the Secretariat of Prison Administration (three penitentiaries, a Provisional Detention Center and a Penitentiary Progression Center) 657 (52.1%) participated in the research. Of the 372 employees of the five CASA Foundations known as “CASA”, 249 (66.9%) chose to participate and of the 511 employees of the two custodial and psychiatric treatment hospitals known as “hospitals”, 153 (29.9%) participated.

In relation to the sociodemographic characteristics of this population the distribution of the employees according to sex, age, marital status, color or race and level of education showed a significant association between the different prison units (p < 0.05). The majority were male, between the ages of 30 and 49 years, married or in a civil union, white, and with high school education. At the hospitals, it should be noted that women represented 50.3% of the employees and that 36.0% of the professionals belonged to the age group 50–59 (Table 1).

**Table 1:** Sociodemographic characteristics of the employees of different types of prison units in the municipality of Franco da Rocha, state of São Paulo, 2015.

Employee characteristics		CASA Foundation	Hospitals	Prison	Total	x^2^	p
		(n = 230)	(n = 230)	(n = 230)	n = 1,059		
		n (%)	n (%)	n (%)	n (%)		
Gender						56.95	**< 0.001**
	Male	171 (68.7)	76 (49.7)	517 (78.7)	764 (72.1)		
	Female	78 (31.3)	77 (50.3)	140 (21.3)	295 (27.9)		
Age group						52.31	**< 0.001**
	18 to 29	30 (12.0)	10 (6.5)	82 (12.5)	122 (11.5)		
	30 to 39	97 (39.0)	40 (26.1)	239 (36.4)	376 (35.5)		
	40 to 49	85 (34.1)	39 (25.5)	217 (33.0)	341 (32.3)		
	50 to 59	35 (14.1)	55 (36.0)	100 (15.2)	190 (17.9)		
	60 or older	2 (0.8)	9 (5.9)	19 (2.9)	30 (2.8)		
Marital status						15.89	**0.014**
	Single	64 (25.7)	39 (25.5)	143 (21.8)	246 (23.2)		
	Married/Civil union	156 (62.7)	92 (60.1)	446 (67.9)	694 (65.5)		
	Separated/Divorced	28 (11.2)	15 (9.8)	60 (9.1)	103 (9.8)		
	Widowed	1 (0.4)	7 (4.6)	8 (1.2)	16 (1.5)		
Ethnic group						36.98	**< 0.001**
	White	128 (51.4)	102 (66.7)	472 (71.8)	702 (66.3)		
	Black/Dark skinned	116 (46.6)	51 (33.3)	181 (27.5)	348 (32.8)		
	Other	5 (2.0)	0	4 (0.7)	9 (0.9)		
Education						3.38	**< 0.001**
	Middle school	8 (3.2)	7 (4.6)	19 (2.9)	34 (3.2)		
	High school	140 (56.2)	85 (55.5)	401 (61.0)	626 (59.1)		
	Higher education	101 (40.6)	61 (39.9)	237 (36.1)	399 (37.7)		

CASA: Centro de Atendimento Socioeducativo ao Adolescente

P value less than or equal to 0.05, in bold, indicating that there are statistically significant differences between the variables compared.

Regarding the clinical-epidemiological characteristics of the employees (Table 2), there was no significant association between coughing, smoking or other lung disease and prison units (p > 0.05).

**Table 2:** Clinical-epidemiological characteristics of employees of different types of prison units in the municipality of Franco da Rocha, state of São Paulo, 2015.

Results		CASA Foundation	Hospitals	Prison	Total	x^2^	p
		(n = 230)	(n = 230)	(n = 230)	n = 1,059		
		n (%)	n (%)	n (%)	n (%)		
Cough						1.75	0.417
	Yes	42 (16.9)	24 (15.7)	89 (13.5)	155 (14.6)		
	No	207 (83.1)	129 (84.3)	568 (86.5)	904 (85.4)		
Smoking						4.51	0.105
	Yes	48 (19.3)	39 (25.5)	118 (18.0)	205 (19.4)		
	No	201 (80.7)	114 (74.5)	539 (82.0)	854 (80.6)		
Other lung disease						5.13	0.077
	Yes	20 (8.0)	9 (5.9)	28 (4.3)	57 (5.4)		
	No	229 (92.0)	144 (94.1)	629 (95.7)	1002 (94.6)		
Previous tuberculosis						107.7	**< 0.001**
	Yes	39 (25.5)	16 (2.4)	47 (18.9)	102 (9.6)		
	No	114 (74.51)	641 (97.6)	202 (81.1)	957 (90.4)		
Contact with detainee						14.12	**< 0.001**
	Yes	141 (92.2)	551 (83.9)	194 (77.9)	886 (83.7)		
	No	12 (7.8)	106 (16.1)	55 (22.1)	173 (16.3)		
Sputum smear microscopy						NA	
	Negative	246 (98.8)	146 (95.4)	220 (33.5)	612 (57.8)		
	Positive	-	-	-	-		
	Culture					NA	
	Negative	246 (98.8)	146 (95.4)	220 (33.5)	612 (57.8)		
	Positive	-	-	-	-		
	Chest X-ray					1.51	0.471
	Normal	18 (11.8)	127 (19.3)	48 (19.3)	193 (18.2)		
	Altered	3 (1.9)	36 (5.5)	9 (3.6)	48 (4.5)		

CASA: Centro de Atendimento Socioeducativo ao Adolescente; NA: not applicable

P value less than or equal to 0.05, in bold, indicating that there are statistically significant differences between the variables compared.

The proportion of employees with previous tuberculosis differed between the units (p < 0.001). In hospitals, 25.5% of employees had previously become ill with TB.

Of the 1,059 employees analyzed, 886 (83.7%) reported in the individual survey that they had direct contact with detainees and this type of contact had different distribution among the different units (p < 0.001). In all of them the majority of employees were contacts and it should be noted that the percentage in the hospitals was 92.2%.

There was no positive test in sputum smear microscopy and culture, meaning that no employee had smear-positive pulmonary TB. It is noteworthy that only 57.8% (612) of the employees agreed to sputum collection for the bacteriology of TB. Many refused, claiming they had no expectoration or any other symptom that would justify collecting the material.

There was no significant association between the results of the chest X-ray and the different prison units (p > 0.05). Of the 241 (22.8%) professionals who performed the exam, 48 (19.9%) presented the following alterations: 11 radiological images suspected of TB; 36 suspected of another pneumopathy and an image with an increased cardiac area. All of them were referred to the Health Units of the region. The 11 employees with radiological images suspected of TB will be the target of a new study.

The tuberculin test was applied and read for 945 (89.2%) professionals. Of these, 797 (84.3%) worked directly with detainees, that is, they were “contacts”, and 148 (15.7%) were “non-contacts”. The main reasons for not performing the tuberculin test in the other 114 (10.8%) employees were the refusal and non-attendance to the application or the reading of the test.

In the evaluation of the sociodemographic and clinical-epidemiological characteristics of the employees in relation to LTBI due to the positive tuberculin test (PPD ≥ 10 mm) we observed that for CASA employees level of education was a factor associated with LTBI (p = 0.018). The other variables did not show any association (Table 3). In the hospitals, the age group was associated with LTBI (p = 0.038), with employees aged 30 to 59 years being more positive to PPD. Employees working in penitentiaries had the following factors associated with LTBI: contact with detainee (p = 0.005); male (p < 0.001); age group (p < 0.001); color or race (p = 0.001) and smoking (p = 0.013).

**Table 3:** Latent tuberculosis infection in a prison staff, according to sociodemographic characteristics, clinical-epidemiological characteristics, and place of work. Franco da Rocha, state of São Paulo, 2015.

Variable		CASA Foundation		Hospitals		Prison	
		PPD < 10	PPD ≥ 10	PPD < 10	PPD ≥ 10	PPD < 10	PPD ≥ 10
		n (%)	n (%)	n (%)	n (%)	n (%)	n (%)
Contact with detainee							
	No	17 (40.5)	25 (59.5)	6 (66.7)	3 (33.3)	78 (80.4)	19 (19.6)
	Yes	89 (55.6	71 (44.4)	59 (48.8)	62 (51.2)	340 (65.9)	176 (34.1)
	p^b^		0.080		0.492		**0.005**
Gender							
	Male	75 (52.4)	68 (47.6)	33 (49.3)	34 (50.7)	318 (64.9)	172 (35.1)
	Female	31 (52.5)	28 (47.5)	32 (50.8)	31 (49.2)	100 (81.3)	23 (18.7)
	p^b^		0.990		0.861		**< 0.001**
Age group							
	18 to 29	12 (54.5)	10 (45.5)	6 (100)	0	67 (89.3)	8 (10.7)
	30 to 39	49 (60.5)	32 (39.5)	14 (43.8)	18 (56.3)	157 (70.7)	65 (29.3)
	40 to 49	31 (41.3)	44 (58.7)	17 (47.2)	19 (52.8)	126 (60.9)	81 (39.1)
	50 to 59	13 (59.1)	9 (40.9)	21 (44.7)	26 (55.3)	58 (61.7)	36 (38.3)
	60 or older	1 (50.0)	1 (50.0)	7 (77.8)	2 (22.2)	10 (66.7)	5 (33.3)
	p^b^		0.181		**0,038** c		**< 0.001**
Marital status							
	Single	28 (52.8)	25 (47.2)	16 (50.0)	16 (50.0)	101 (76.5)	31 (23.5)
	Married/Civil union	68 (54.8)	56 (45.2)	39 (49.4)	40 (50.6)	276 (66.7)	138 (33.3)
	Separated/Divorced	9 (37.5)	15 (62.5)	6 (50.0)	6 (50.0)	37 (62.7)	22 (37.3)
	Widowed	1 (100)	0	4 (66.7)	2 (33.3)	4 (50.0)	4 (50.0)
	p^b^		**0,313** c		**0,913** c		0.082
Ethnic group							
	White	59 (56.7)	45 (43.3)	42 (50.0)	42 (50.0)	319 (72.3)	122 (27.7)
	Black/Dark skinned	46 (49.5)	47 (50.5)	23 (50.0)	23 (50.0)	95 (56.9)	72 (43.1)
	Other	1 (20.0)	4 (80.0)	0	0	3 (75.0)	1 (25.0)
	p^b^		**0,202** c		1		**0.001** c
Education level							
	Middle school	0	5 (100)	5 (71.4)	2 (28.6)	10 (62.5)	6 (37.5)
	High school	63 (58.3	45 (41.7)	37 (48.7)	39 (51.3)	250 (66.1)	128 (33.9)
	Higher education	43 (48.3)	46 (51.7)	23 (48.9)	24 (51.1)	157 (72.0)	61 (28.0)
	p^b^		**0,018** c		**0,589** c		0.295
Cough							
	No	84 (51.2)	80 (48.8)	56 (50.5)	55 (49.5)	365 (68.5)	168 (31.5)
	Yes	22 (57.9)	16 (42.1)	9 (47.4)	10 (52.6)	53 (66.3)	27 (33.8)
	p^b^		0.458		0.804		0.690
Smoking							
	No	90 (54.2)	76 (45.8)	50 (52.6)	45 (47.4)	352 (70.4)	148 (29.6)
	Yes	16 (45.7)	19 (54.3)	15 (42.9)	20 (57.1)	66 (58.4)	47 (41.6)
	p^b^		0.360		0.323		**0.013**
Other lung disease							
	No	99 (52.7)	89 (47.3)	60 (49.6)	61 (50.4)	398 (68.3)	185 (31.7)
	Yes	7 (50.0)	7 (50.0)	5 (55.6)	4 (44.4)	20 (66.7)	10 (33.3)
	p^b^		0.848		1^c^		0.854

CASA: Centro de Atendimento Socioeducativo ao Adolescente; PPD: Purified Protein Derivative

a Result of the tuberculin test, according to the PPD result, with a cut-off point of 10 mm.

b Pearson's chi-squared test.

c Fisher's exact test.

P value less than or equal to 0.05, in bold, indicating that there are statistically significant differences between the variables compared.

In the analysis of the factors associated with LTBI in the employees who worked in penitentiaries (Table 4), the OR was calculated by means of multiple logistic regression. Staff in contact with prisoners are 2.12 times more likely to present LTBI, compared to those who do not have contact (p = 0.008). Additionally, male employees were almost twice as likely to be infected compared to female employees (OR = 1.97, 95%CI 1.19–3.27). Employees between 30 and 39 years old, 40 to 49 years old, and 50 to 59 years old presented a greater chance of infection compared to employees of up to 29 years old. Those aged 40 to 49 years old were more than four times more likely to be infected (OR = 4.32, 95%CI 1.94–9.60). Other factors related to LTBI were non-white color or race (OR = 1.89, 95%CI 1.29–2.78) compared to employees of other colors or races, as were those who smoked, whose chance of being infected was 1.64 times compared to those who did not smoke (p = 0.029).

**Table 4:** Sociodemographic and clinical-epidemiological factors associated with latent tuberculosis infection in prison staff. Franco da Rocha, state of São Paulo, 2015.

Variable		Unajusted PR	p	Adjusted PR^b^	p
		(95%CI)		(95%CI)	
Contact with detainee					
	No	1		1	
	Yes	2.13 (1.25–3.62)	0.006	2.12 (1.21–3.71)	**0.008**
Gender					
	Female	1		1	
	Male	2.35 (1.44–3.84)	0.001	1.97 (1.19–3.27)	**0.009**
Age group					
	18 to 29	1		1	
	30 to 39	3.47 (1.58–7.63)	0.002	2.98 (1.34–6.63)	**0.008**
	40 to 49	5.38 (2.46–11.80)	< 0.001	4.32 (1.94–9.60)	**< 0.001**
	50 to 59	5.20 (2.24–10.08)	< 0.001	3.98 (1.68–9.43)	**0.002**
	60 or older	4.19 (1.14–15.37)	0.031	2.90 (0.75–11.12)	0.122
Marital status					
	Single	1			
	Married/Civil union	1.63 (1.04–2.56)	0.034		
	Separated/Divorced	1.94 (1.00–3.76)	0.051		
	Widowed	3.26 (0.77–13.80)	0.109		
Ethnic group					
	White	1		1	
	Non-white	1.95 (1.35–2.81)	< 0.001	1.89 (1.29–2.78)	**0.001**
Education level					
	Middle school	1			
	High school	0.85 (0.30–2.40)	0.764		
	Higher education	0.65 (0.23–1.86)	0.648		
Cough					
	No	1			
	Yes	1.11 (0.67–1.82)	0.690		
Smoking					
	No	1		1	
	Yes	1.69 (1.11–2.58)	0.014	1.64 (1.05–2.55)	**0.029**
Other lung disease					
	No	1			
	Yes	1.08 (0.49–2.34)	0.854		

a Positive tuberculin test (PPD ≥ 10 mm).

b Hosmer-Lemeshow test to adjust the model x^2^ = 3.15; p = 0.925, under the null hypothesis that the model is good.

P value less than or equal to 0.05, in bold, indicating that there are statistically significant differences between the variables compared.

## DISCUSSION

The main reasons for the low adherence to the survey, since only 49.4% of the employees were present and accepted to participate in the study, were the difficulty in reconciling the work schedule of the employees with the schedule of the activities of the members of the research, vacations, medical licenses and outside activities. Others refused to participate or did not attend the application or the reading of the tuberculin test. There were greater adherence and interest of the professionals who had contact with the detainees, of whom 83.7% agreed to carry out all the exams. The factor that explains the association between being in contact with detainees and LTBI is the greater exposure to the bacilli to which they are subjected. The professionals who work close to the inmates are at greater risk of becoming infected with *M. tuberculosis* and become ill with TB^8^ ^,^ ^11^ ^,^ ^12^.

Due to the different characteristics of the prison units analyzed there was an association between the professional being the contact of detainees and the LTBI only in the penitentiaries. In fact the other units have characteristics that minimize the risk of transmission of the disease. In the units of the CASA Foundation there is no overcrowding of adolescents. Cells are well ventilated, confinement time is shorter and adolescents represent a lower risk group for tuberculosis. In hospitals, there are no cells but well-ventilated wards. Despite belonging to groups at high risk for TB, inmates and mental patients spend the day outdoors, in large spaces of leisure, using the wards only to sleep. Those who present other pathologies, besides mental illness, are in separate rooms. However, we noted that 25.5% of hospital staff had previously become ill with TB, possibly because 36% of them were over 50 years of age and that the age group of 30 to 59 years old was associated with LTBI.

In order to demonstrate that prison employees are more likely to be infected and sick with TB, Steenland et al.^27^ studied tuberculin turnovers in prisoners in New York state after an outbreak of TB in prisoners and found that 33% of the new turns were due to occupational exposure. In a study at a women's prison in Montreal, Canada, Jochem et al.^13^ found a positive association between working in prisons and tuberculosis infection. Nogueira et al.^18^ showed that 62.4% of the employees that worked directly with the detainees, in two penitentiaries in the state of São Paulo, were infected by the TB bacillus. Al-Darraji et al.^2^ found a prevalence of LTBI in 81% of employees of a maximum security prison in Malaysia.

The percentage of detainee contact staff who were infected in this study was lower than that found in the studies by Nogueira et al.^18^ and Al-Darraji et al.^2^. This may have occurred because, apart from the different characteristics of the prison units studied, the sanitary conditions of the prisons of Franco da Rocha were very good.

This study's limitations are the fact that it provided a static view of the problem since the collection of data on both the exposure and the outcome occurred simultaneously making it difficult to understand the temporal relationship between them. In addition, the tuberculin test has its own limitations regarding the application and reading of the test and the possibility of false or negative results even though it is still considered the most important exam for the diagnosis of LTBI in Brazil. Finally, the small number of national and international studies with the same objective and with the same target population limited the comparison of the results obtained in this study.

There are several risk factors for LTBI and active TB such as: sex; age; low socioeconomic status; low level of education; smoking; HIV/TB coinfection; use of alcohol and other drugs; malnutrition; diabetes mellitus; renal failure and other metabolic conditions; use of corticosteroids, other immunosuppressants and chemotherapy; among others. These factors are exacerbated in a confinement environment with overcrowding, poor ventilation and lighting and low hygiene standards such as Brazilian prisons^5^ ^,^ ^7^ ^,^ ^9^ ^,^ ^16^ ^,^ ^17^ ^,^ ^23^ ^,^ ^29^ ^,^ ^b^ ^,^ ^i^.

In this study, the factors associated with LTBI in prison staff were in addition to having contact with detainees, male, in the age group of 30 to 59 years old, of non-white color or race and smoker.

It is known that the prevalence of TB is higher in male adults in the proportion of 2:1 to 3:1 in relation to women, probably due to habits and behaviors more related to the man and that also interact in the infection by *M. tuberculosis*, such as alcohol and tobacco abuse, drug use and occupational exposure to inhalant offenders. In addition, men access health services less reducing the chance of early diagnosis of TB^28^ ^,^ ^31^ ^,^ ^b^ ^,^ ^o^. In 2012, the male gender had an incidence coefficient of 50.2/100,000 inhabitants. (2.1 times greater than that of females)^p^.

There are big differences in the incidence of TB related to age. In less developed countries, the most affected population is young adults reflecting a recent transmission. The disease is more frequent in the age group between 25 and 34 years and the highest incidence rate occurs in the range between 45 and 54 years of age^31^ ^,^ ^o^.

In relation to color or race black and dark-skinned people are at higher risk of becoming ill or dying of tuberculosis when compared to white people. In Brazil, 14.1% of the black or darkskinned population is among the poorest 10% of the country. Social vulnerability possibly justifies the increased risk of becoming ill or dying from TB^q^.

Smoking has been identified as a risk factor for TB since 1918. A systematic review (conducted by WHO and the International Union Against Tuberculosis and Lung Diseases, The Union) confirmed the association between tobacco use and TB infection, TB disease, TB recurrence and disease mortality as tobacco alters the mucociliary function and filtration of inhaled substances promoting the adhesion of bacteria to the epithelial cells of the airways increasing alveolar permeability and reducing humoral immunity mediated by cells^14^ ^,^ ^16^ ^,^ ^20^ ^,^ ^28^ ^,^ ᵃ ^,^ ^i^. In the study by Al-Darraji et al.^2^, LTBI was associated with the employee's time working in a prison in Malaysia and with smoking. Those who smoked had 1.9 times the chance of being infected; result similar to that found in this study.

Considering the high risk of TB in prisons, the State Health Department of São Paulo^c^, the World Health Organization^9^ ^,^ ^30^ and the Centers for Disease Control and Prevention (CDC)^6^ recommend greater educational and preventive control for professionals working with detainees, such as health professionals, guards, teachers, etc. If actions to confront TB in prisons are not effective it will be unfeasible to control it in the community^9^ ^,^ ^17^ ^,^ ^30^.

The STOP TB Strategy of the World Health Organization aims at “a world free of tuberculosis” and one of the targets is to eliminate TB as a public health problem (one case per million inhabitants) by 2050^9^ ^,^ ^31^.

This study suggests that prison staff, especially those working in penitentiaries and who have contact with detainees, should receive adequate TB prevention and control training by participating in an occupational health program with periodic testing for infection and for the disease. The LTBI investigation should be performed in the admission and periodic examinations (annually) through the tuberculin test. If they are infected they should have access to LTBI treatment. Employees with signs or symptoms consistent with active TB should seek medical assistance and undergo laboratory tests and chest X-rays.

Due to their high risk of becoming infected and ill with TB prison staff should be given special attention by health authorities and included in the public policy agenda. In addition, smoking control could be integrated into the TB control program in prisons.

The results of this study can contribute to the creation of a proposal of occupational health in tuberculosis for professionals of the prison system.

## Footnotes

a Ministério da Saúde (BR), Secretaria de Vigilância em Saúde, Departamento de Vigilância Epidemiológica. Manual de recomendações para o controle da tuberculose no Brasil. Brasília (DF); 2011 [cited 2016 Aug 16]. (Série A. Normas e Manuais Técnicos). Available from: http://bvsms.saude.gov.br/bvs/publicacoes/manual_recomendacoes_controle_tuberculose_brasil.pdf

b Sacchi FPC. Fatores de risco para tuberculose em população indígena, carcerária e urbana de Dourados-MS [dissetation]. Dourados: Universidade Federal da Grande Dourados; 2011.

c São Paulo. Secretaria de Estado da Saúde, Coordenação dos Institutos de Pesquisa, Centro de Vigilância Epidemiológica “Prof. Alexandre Vranjac”, Divisão de Tuberculose e outras Pneumopatias. Recomendações para o controle da tuberculose nas prisões. São Paulo; 1999.

d Souza J. Sistema carcerário brasileiro: a ineficiência, as mazelas e o descaso presentes nos presídios superlotados e esquecidos pelo poder público. In: Jusbrasil [cited 2015 Jul 18]. Available from: http://jucienesouza.jusbrasil.com. br/artigos/129905259/sistema-carcerario-brasileiro

e Ministério da Justiça (BR), Departamento Penitenciário Nacional. Levantamento Nacional de Informações Penitenciárias INFOPEN - junho de 2014 [cited 2016 Aug 16]. Available from: http://www.justica.gov.br/noticias/mj-divulgara-novo-relatorio-do-infopen-nesta-terca-feira/relatorio-depen-versao-web.pdf

f Secretaria da Administração Penitenciária do Estado de São Paulo. 165 Unidades Prisionais. São Paulo; s.d. [cited 2016 Aug 30]. Available from: http://www.sap.sp.gov.br

g São Paulo. Secretaria de Estado da Justiça e da Defesa da Cidadania. CASA Foundation - Centro de Atendimento Socioeducativo ao Adolescente. São Paulo; s.d. [cited 2016 Aug 16]. Available from: http://www.fundacaocasa.sp.gov.br

i Feitosa IMLC. Infecção latente por tuberculose em Teresina, Piauí, Brasil: uma análise dos casos indicados para o tratamento preventivo [dissertation]. Rio de Janeiro: Escola Nacional de Saúde Pública Sergio Arouca; 2015.

j Sousa BA, Braz VHC, Paes ALV. Prevalência de infecção por tuberculose em funcionários de um hospital universitário. [cited 2015 Jul 18]. Available from: http://files.bvs.br/upload/S/0101-5907/2011/v25n1/a2587.pdf

k Ministério da Saúde (BR), Secretaria de Vigilância em Saúde, Departamento de Vigilância Epidemiológica. Manual nacional de vigilância laboratorial da tuberculose e outras micobactérias. Brasília (DF); 2008 [cited 2016 Aug 16]. (Série A. Normas e Manuais Técnicos). Available from: http://bvsms.saude.gov.br/bvs/publicacoes/manual_vigilancia_laboratorial_tuberculose.pdf

l Ministério da Saúde (BR), Secretaria de Vigilância em Saúde, Departamento de Vigilância das Doenças Transmissíveis. Técnicas de aplicação e leitura da prova tuberculínica. Brasília (DF); 2014 [cited 2016 Aug 16]. Available from: http://bvsms.saude.gov.br/bvs/publicacoes/tecnicas_aplicacao_leitura_prova_tuberculinica.pdf

m Abrahão RMCM. Diagnóstico da tuberculose na população carcerária dos Distritos Policiais da Zona Oeste da Cidade de São Paulo [these]. São Paulo: Faculdade de Saúde Pública; 2004. https://doi.org/10.11606/T.6.2004.tde-03032011-144652

n Centers for Disease Control and Prevention, Division of Tuberculosis Elimination. Mantoux Tuberculosis Skin Test facilitator guide: appendix D: Mantoux Tuberculin Skin Test interpretation table. Atlanta: CDC; 2012 [cited 2016 Aug 16]. Available from: http://www.heartlandntbc.org/assets/training/mini-fellowship/PediatricToolBox/CDC/ed_training/publications/mantoux/appendix_D.htm

o Ministério da Saúde (BR). Secretaria de Vigilância em Saúde. Especial Tuberculose. *Bol Epidemiol*. 2012;43:1-12.

p Ministério da Saúde (BR), Secretaria de Vigilância em Saúde. O controle da tuberculose no Brasil: avanços, inovações e desafios. *Bol Epidemiol*. 2014;45(2):1-13.

q Ministério da Saúde (BR), Secretaria de Vigilância em Saúde. Indicadores de Vigilância em Saúde, analisados segundo a variável raça/cor. *Bol Epidemiol*. 2015;46(10):1-13.
